# Association between critical care admission and chronic medication discontinuation post-hospital discharge: A retrospective cohort study

**DOI:** 10.1177/17511437241230260

**Published:** 2024-03-01

**Authors:** Charvi Kanodia, Richard S Bourne, Elizabeth T Mansi, Nazir I Lone

**Affiliations:** 1 Edinburgh Medical School, University of Edinburgh, Edinburgh, UK; 2 Departments of Pharmacy and Critical Care, Sheffield Teaching Hospitals NHS Foundation Trust, Sheffield, UK; 3 Division of Pharmacy and Optometry, Faculty of Biology, Medicine and Health, School of Health Sciences, The University of Manchester, Manchester, UK; 4 Usher Institute, University of Edinburgh, Edinburgh, UK

**Keywords:** Transitions of care, critical care, chronic medications, discontinuation

## Abstract

*Background:* Discontinuation of important chronic medication after hospitalisation is common. This study aimed to investigate the association between critical care (vs non-critical care) admission and discontinuation of chronic medications post-hospital discharge, along with factors associated with discontinuation among critical care survivors. *Methods:* This was a retrospective cohort study in Lothian, Scotland of adults who were admitted to hospital between 01/01/2012 and 31/12/2019 and survived to hospital discharge. Medication classes investigated were statins, angiotensin converting enzyme inhibitors/angiotensin receptor blockers (ACEi/ARBs), beta-blockers, oral anticoagulants, and thyroid hormones. The risk of medication discontinuation for each class was estimated by odds ratios (OR), with 95% confidence intervals (95%CI), using multivariable logistic regression adjusted for patient demographics, main clinical condition, and index comorbidity. A secondary analysis assessed factors associated with discontinuation in critical care survivors. *Results:* There were 22,340 critical care and 367,185 non-critical care survivors included. Critical care admission had the highest association with ACEi/ARBs discontinuation (adjusted OR 2.41, 95%CI: 2.26–2.58), followed by oral anticoagulants (adjusted OR 1.33, 95%CI: 1.15–1.53), and beta blockers (adjusted OR 1.18, 95%CI: 1.07–1.29). There was no significant association with thyroid hormones or statin discontinuation. Among critical care survivors, hospital length of stay of 14 days or more was associated with increased discontinuation across all medication classes. *Conclusion:* Critical care admission was associated with discontinuation of three out of five medication classes studied (ACEi/ARBs, beta-blockers, and oral anticoagulants). Further research is needed to understand the reason for increased medication discontinuation in critical care survivors and how these risks can be mitigated to improve patient outcomes.

## Introduction

With increasing survival rates of complex critical care patients, there is interest and new understanding of the complications and morbidity survivors experience.^1,2^ These are multifactorial and increase healthcare resources required by survivors after hospital discharge.^3,4^ Unplanned hospital readmission rates are high, being influenced by acute, and pre-existing illnesses.^5^ Medication-related problems are an important factor, with the presence and extent of preadmission polypharmacy independently associated with patient emergency readmission.^6^ Critical care survivors have high rates of medication-related problems, being reported in up to 80% of patients post hospital discharge.^7^ These medication-related problems are comprised of continuation of potentially inappropriate acute medication (e.g. sedatives, opioids) and omission of important chronic medication (e.g. cardiovascular medicines), being compounded by medication errors.^7^ Survivors of critical illness are particularly vulnerable to medication-related problems due to the sequalae of post-intensive care syndrome and residual impaired organ function.^1,8^ The prevalence of multimorbidity is twice as high in critical care patients than non-critical care patients,^9^ underlining the potential impact inappropriate discontinuation of chronic medications may have on the poor health outcomes in critical care survivors post-hospital discharge.^5^

Previous research suggests an association between critical care admission and potentially inappropriate discontinuation of chronic medications after hospital discharge. In a population-based cohort study conducted in Canada, Bell et al.^10^ found critical care patients prescribed chronic medications had a higher risk of discontinuation than non-critical care patients. However, this was restricted to older patients (⩾66 years) and only included hospitalisations ⩽15 days, potentially obscuring the effect for younger or more severely ill patients. Several features of critical patient care contribute to higher chronic medication discontinuation rates. These include an acute care focus, additional transitions of care, and the routine requirement to discontinue certain medicines during critical illness based on clinical status.^11–13^ Long term outcomes from chronic diseases are not the primary focus during critical care,^14^ and some medicines may be inappropriate during critical care initially (e.g. antihypertensives), leading them to be discontinued and potentially not restarted after appropriate recovery.^15^ Care of the critically ill patient is complex, requiring effective interactions, communication, and collaboration of care teams in a dynamic and fast-paced clinical environment. Medication safety and continuity on transition in patient care are vulnerable to system and process complexities in addition to fluctuations in patient illness and recovery.^16,17^

Given the limitations of previous research, our primary aim was to estimate the association between critical care admission and discontinuation of chronic medications in adult hospitalised patients. We chose four common cardiovascular medication classes (statins, angiotensin converting enzyme inhibitors/angiotensin receptor blockers (ACEi/ARBs), beta-blockers, and oral anticoagulants), as well as thyroid hormones (of which discontinuation is rarely indicated),^10,18^ to investigate this relationship. Our secondary aim was to identify patient factors associated with discontinuation of chronic medication among critical illness survivors.

## Methods

### Study design, setting, and participants

This study was a retrospective cohort study design based on a larger study of patients who attended an emergency department (ED) in one of the three acute hospitals in Lothian, Scotland at least one time during the period 01/01/2012–31/12/2019. The datasets used were from the following: Scottish Morbidity Record Inpatient (SMR01, acute hospitalisations), Scottish Intensive Care Society Audit Group (SICSAG), Prescribing Information System (PIS, community prescribing), and National Records of Scotland (NRS, death records). These databases are high in quality, with SICSAG being the national benchmarking audit for critical care in Scotland. The data were pseudonymised and linked before release to researchers. Data were stored and analysed in a Safe Haven environment.

The full patient population consisted of patients aged 18 years or older who both attended a Lothian ED at one point during the study period and were admitted to hospital between 01/01/2012 and 31/12/2019, surviving to discharge. Patients with missing sex were excluded (<0.01%). For patients with multiple hospital admissions, one admission was selected at random so that each patient was represented only once. From this full study population, patients were included in each medication category if they were “chronic users” at risk for discontinuation. Chronic users were defined as receiving two or more prescriptions within 180 days prior to hospitalisation in the medication class of interest.

### Chronic medication classes of interest

The medication classes of interest were defined by their British National Formulary (BNF) chapters and included the following: (1) thyroid hormones (BNF Chapter 6.2.1); (2) statins (2.12 lipid regulating drugs); (3) angiotensin converting enzyme inhibitor (ACEi) and angiotensin receptor blocker (ARB) medication (2.5.5 renin-angiotensin system drugs); (4) beta-blockers (2.4 beta-adrenoceptor blocking drugs); and (5) oral anticoagulants (2.8.2).^19^ The patient groups for the five medication classes were not mutually exclusive, that is, it was possible for a patient to be in both the thyroid and statin group, provided they were prescribed both medications two or more times in the 180 days prior to hospital admission.

Medication classes were chosen to reflect important evidence-based chronic medicines vulnerable to discontinuation after a critical care patient episode, with potential negative impact on patient recovery and outcomes. Selected cardiovascular medicines have previously been identified as high risk for discontinuation after a critical care patient episode^10^ and antihypertensives are routinely discontinued on critical care admission in patients with shock states and/or acute kidney injury. Thyroid hormones have previously been reported to have high discontinuation rates in critical care survivors.^10^ Having few contra-indications, their continuation has been proposed as an indicator of medicines reconciliation processes in critical care units.^20^

### Exposure

The primary exposure was critical care admission, which was defined by admission to intensive care or high-dependency unit. For the primary analysis, this was treated as a binary variable (critical care admission vs hospitalised non-critical care). For the secondary analysis, characteristics of only those who received critical care were considered as potential factors for medication discontinuation.

### Outcome

The primary outcome was absence of a recorded community prescription for the medication class of interest within 90 days of hospital discharge (“discontinuation of chronic medication”).

### Confounders

Patient demographic characteristics included sex, age group, ethnicity, and socioeconomic status (as measured by Scottish index of multiple deprivation (SIMD) quintile). SIMD is a relative measure of deprivation using seven domains: income, employment, education, health, access to services, crime, and housing.^21^ Main condition at hospitalisation was based on International Classification of Diseases – Tenth Edition (ICD-10) codes and categorised into one of the following disorders: Neoplasms (C00-D49), Circulatory (I00-I99), Respiratory (J00-J99), Digestive (K00-K95), Symptoms, signs and abnormal clinical and laboratory findings not classified elsewhere (R00-R99), Injury or poisoning (S00-T88), and all other conditions. Comorbidity categories were derived from ICD-10 diagnosis codes from hospitalisations in the 5 years prior to the index hospitalisation. This was based on the Elixhauser comorbidity index,^22^ which categorises comorbidities based on ICD codes in a dichotomous manner. Patients were categorised as having 0, 1, 2, or 3 or more comorbidities.

Several additional characteristics were considered in the secondary analysis to assess patient factors associated with chronic medication discontinuation amongst critical illness survivors. These were the Acute Physiology and Chronic Health Evaluation (APACHE II) Score (in categories of 0, 1–8, 9+), length of hospital stay (<7, 7–13, 14+ days), ventilatory support (none, non-invasive, invasive), cardiovascular support (binary), renal support (binary), highest level of critical care support received (none, level 1, level 2, level 3),^23^ and surgical status (none, elective, emergency).

### Statistical analysis

The data were cleaned and analysed using *R* version 4.2.2. Most of the variables were categorised and reported as counts with percentages. Patients with missing SIMD data were excluded from multivariable analyses while we employed a missing category approach for patients with missing ethnicity.

Logistic regression was performed to estimate the association between critical care admission and medication discontinuation for each of the drug classes, providing unadjusted and adjusted odds ratios (ORs) with 95% confidence intervals (95%CI). Confounders included in the multivariable model were sex, age group, SIMD, ethnicity, main condition at hospitalisation and comorbidity count.

We aimed to identify factors for medication discontinuation among patients with a critical care admission using multivariable logistic regression. This was performed for each medication class and forest plots (showing adjusted ORs) were plotted for covariates in the models (listed above).

### Additional and sensitivity analyses

In an additional analysis, we evaluated the likelihood of discontinuation of all medications for patients taking two or more medications, and compared this between critical care and non-critical care groups. This is because this may be more likely to indicate an unintentional discontinuation of medications. In the sensitivity analysis, patients who died or were readmitted to hospital within 90 days from index discharge were removed from the primary analyses. This was because patients who died or were readmitted to hospitals would not have received community prescriptions and would potentially be misclassified as discontinuers.

### Approvals

The National Health Service (NHS) Lothian/University of Edinburgh Dataloch Project Review and Advisory Committee approved access to this data (ref. SH2019-008). This group ensures information governance, ethical research, and confidentiality for research conducted using NHS Lothian anonymised patient data.

## Results

### Participants

The total critical care population consisted of 22,340 adults, and the total non-critical care population consisted of 367,185 adults. Compared to non-critical care survivors, the critical care survivors had a greater proportion of males (56% vs 47%), and a higher median age (62 vs 56 years). The distribution of SIMD and ethnicity were similar among both groups. The most common main condition category at index hospitalisation for the critical care group was circulatory (20%) and for the non-critical care group, injury (17%) (Table 1).

**Table 1.** Characteristics of adults hospitalised in Lothian who survived to hospital discharge (2012–2019).

Patient characteristics	Critical care *n* = 22,340 (%)	Non-critical care *n* = 367,185 (%)
Sex		
Male	13,117 (56.2)	174,017 (47.4)
Female	10,223 (43.8)	193,168 (52.6)
Age		
Median (IQR)	62.0 (48.0–73.0)	56.0 (38.0–74.0)
Age group		
18–29	1762 (7.5)	54,479 (14.8)
30–39	1893 (8.1)	43,388 (11.8)
40–49	2733 (11.7)	47,712 (13.0)
50–59	4021 (17.2)	54,201 (14.8)
60–69	5171 (22.2)	52,816 (14.4)
70–79	5069 (21.7)	54,771 (14.9)
80+	2691 (11.5)	59,818 (16.3)
Socioeconomic status (SIMD quartile)		
5 (least deprived)	5400 (23.1)	88,649 (24.1)
4	3898 (16.7)	64,330 (17.5)
3	4238 (18.2)	65,883 (17.9)
2	5853 (25.1)	88,973 (24.2)
1 (most deprived)	3713 (15.9)	54,953 (15.0)
Missing	238 (1.0)	4397 (1.2)
Ethnicity		
Asian	274 (1.2)	5755 (1.6)
Black	116 (0.5)	1864 (0.5)
Mixed/other	87 (0.4)	2037 (0.6)
White	19,686 (84.3)	305,034 (83.1)
Missing	3177 (13.6)	52,495 (14.3)
Main condition at hospitalisation		
Digestive	3455 (14.8)	36,153 (9.8)
Circulatory	4729 (20.3)	35,202 (9.6)
Respiratory	2273 (9.7)	29,280 (8.0)
Abnormal findings^a^	724 (3.1)	51,402 (14.0)
Neoplasms	3995 (17.1)	14,848 (4.0)
Injury	3704 (15.9)	62,861 (17.1)
Other	4460 (19.1)	137,439 (37.4)
Index morbidity count		
None	10,986 (47.1)	305,371 (83.2)
1	4193 (18.0)	29,285 (8.0)
2	3318 (14.2)	16,315 (4.4)
3 or more	4843 (20.7)	16,214 (4.4)
Length of hospital stay (days)		
Median (IQR)	9.0 (5.0–19.0)	1.0 (0.0–4.0)
Year discharged		
2012	2682 (11.5)	45,459 (12.4)
2013	2889 (12.4)	44,693 (12.2)
2014	3082 (13.2)	46,459 (12.7)
2015	2947 (12.6)	45,578 (12.4)
2016	2882 (12.3)	42,689 (11.6)
2017	2934 (12.6)	43,888 (12.0)
2018	2980 (12.8)	44,768 (12.2)
2019	2820 (12.1)	52,879 (14.4)
2020	124 (0.5)	772 (0.2)
Hospital readmission within 90 days of hospital discharge	7691 (33.0)	60,692 (16.5)
Died within 90 days of hospital discharge	772 (3.3)	12,705 (3.5)

IQR: interquartile range; SIMD: Scottish Index of Multiple Deprivation.

a Abnormal findings: International Classification of Diseases-10 codes R00-R99 (“Symptoms, signs and abnormal clinical and laboratory findings, not elsewhere classified”).

**Figure 1.**
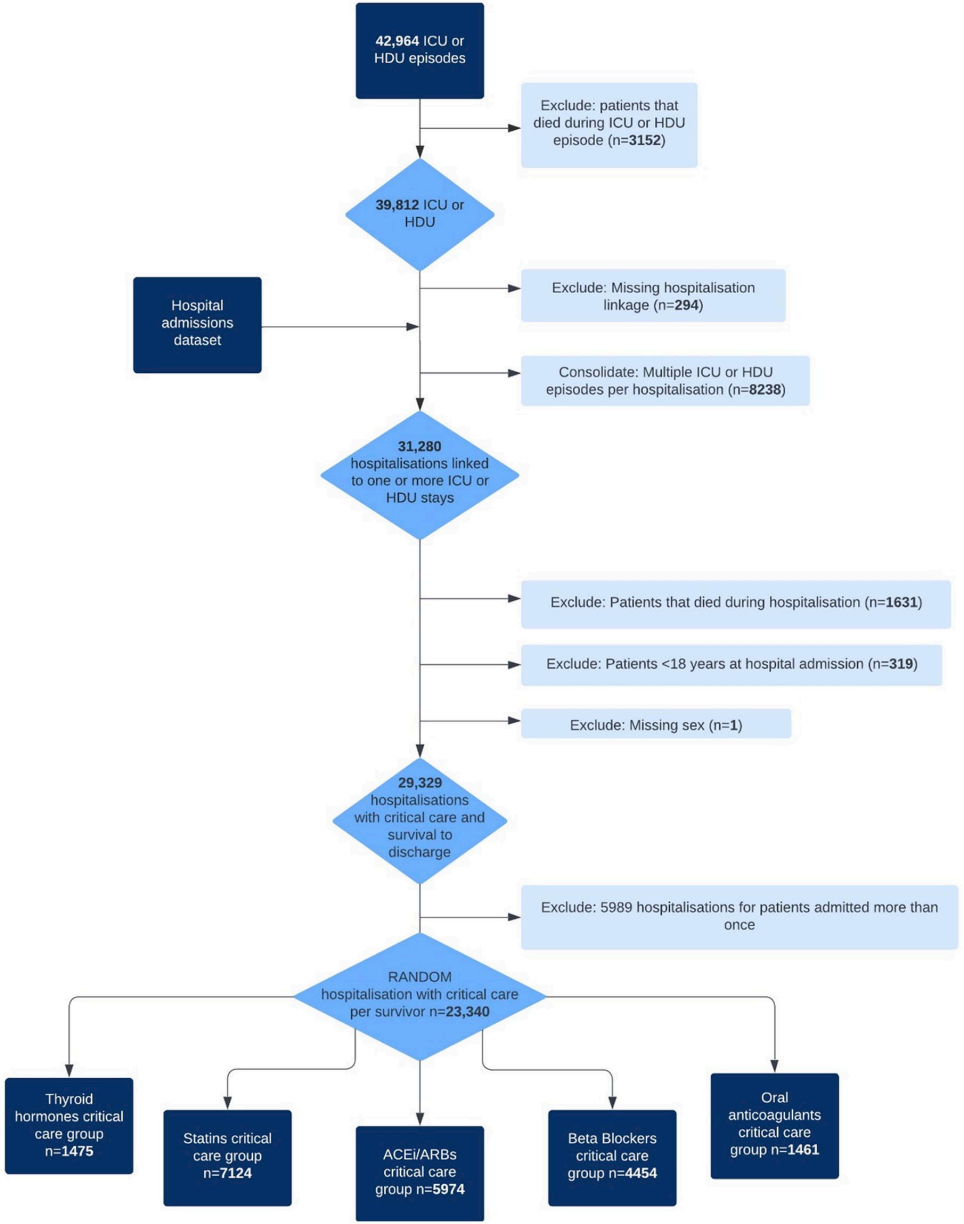
Critical care cohort flow diagram.

**Figure 2.**
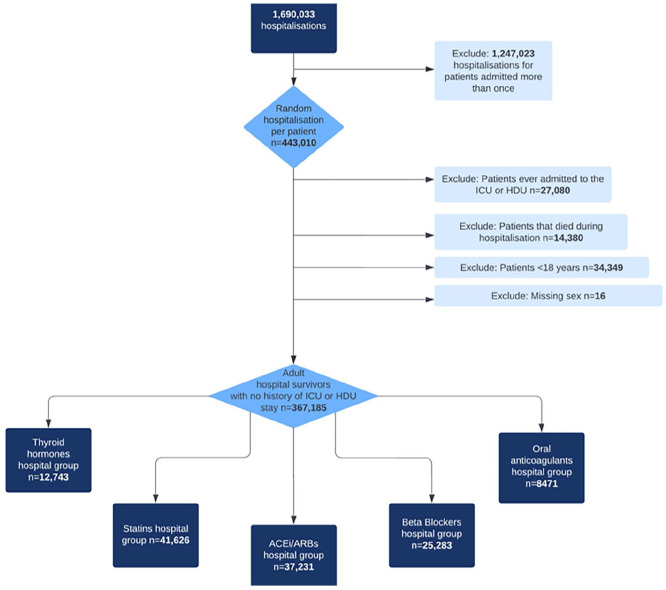
Non-critical care cohort flow diagram. ICU: intensive care unit; HDU: high dependency unit; ACEi: angiotensin converting enzyme inhibitor; ARB: angiotensin receptor blocker.

Among the critical care group, there were 1475 (6.6%) thyroid hormones, 7124 (31.9%) statins, 5974 (26.7%) ACEi/ARB, 4454 (19.9%) beta-blocker, and 1461 (6.5%) anticoagulant chronic users (Figure 1). Among the non-critical care group, there were 12,743 (3.5%) thyroid hormones, 41,626 (11.3%) statin, 37,231 (10.1%) ACEi/ARB, 25,283 (6.9%) beta-blocker, and 8471 (2.3%) anticoagulant chronic users (Figure 2).

### Patient characteristics by medication groups

The thyroid hormones group had the largest proportion of females (74% critical care and 83% non-critical care). Patients from each medication class were older than the overall populations from which they were derived (Table 1). Characteristics for patients in each medication class can be found in Supplemental Tables S1 to S5.

### Outcomes

#### Primary outcomes

Thyroid hormones and statin medication classes both showed the lowest proportion of discontinuation after hospital discharge for both critical care and non-critical care patients (at 7.9% vs 7.6% for thyroid and 14% vs 13% for statins). When confounders were adjusted for, there was no association between critical care and discontinuation of these medication classes (thyroid hormones: adjusted OR 0.97; 95%CI: 0.78–1.19 and statins: adjusted OR 1.06; 95%CI: 0.98–1.14).

ACEi/ARBs, beta blockers, and oral anticoagulants showed higher levels of discontinuation in their drug classes for critical care compared to non-critical care. The association was strongest for ACEi/ARBs: after adjusting for confounders, the risk of discontinuation was more than two-fold for critical care patients compared to non-critical care patients (adjusted OR 2.41; 95%CI: 2.26–2.58). There was a small but significant increased risk of discontinuation in beta-blockers and oral anticoagulants in critical care compared to non-critical care survivors (Table 2).

**Table 2.** Association of critical care and discontinuation of chronic medication by class.

Medication class	Number of discontinuers/chronic critical care users (%)	Number of discontinuers/chronic non-critical care users (%)	Unadjusted OR for discontinuation (95%CI, *p*-value)	Adjusted^a^ OR for discontinuation (95%CI, *p*-value)
Thyroid hormones	116/1475 (7.9)	969/12,743 (7.6)	1.04 (0.84–1.26, *p* = 0.722)	0.97 (0.78–1.19, *p* = 0.747)
Statins	1030/7124 (14)	5446/41,626 (13)	1.12 (1.04–1.21, *p* = 0.002)	1.06 (0.98–1.14, *p* = 0.153)
ACEi/ARBs	1986/5974 (33)	6403/37,231 (17)	2.40 (2.26–2.55, *p* < 0.001)	2.41 (2.26–2.58, *p* < 0.001)
Beta-blockers	784/4454 (18)	4096/25,283 (16)	1.10 (1.02–1.20, *p* = 0.020)	1.18 (1.07–1.29, *p* < 0.001)
Oral anticoagulants	353/1461 (24)	1578/8471 (19)	1.39 (1.22–1.59, *p* < 0.001)	1.33 (1.15–1.53, *p* < 0.001)

OR: odds ratio; CI: confidence interval; ACEi: angiotensin-converting enzyme inhibitor; ARB: angiotensin receptor blocker.

a Adjusted models were adjusted for sex, age group, socioeconomic status, ethnicity, main condition at hospitalisation and comorbidity category.

#### Additional and sensitivity analyses

Restricting the study population to patients who were chronic users of two or more medication classes (*n* = 43,226), 2805 (6.5%) had all of their medications discontinued, which was higher in critical care survivors (467/6441 (7.3%) critical care vs 2338/36,785 (6.4%) non-critical care survivors). However, after adjusting for confounders, critical care admission was not significantly associated with discontinuation of all medications (adjusted OR: 1.05 (0.94–1.17, *p* = 0.379)).

**Figure 3.**
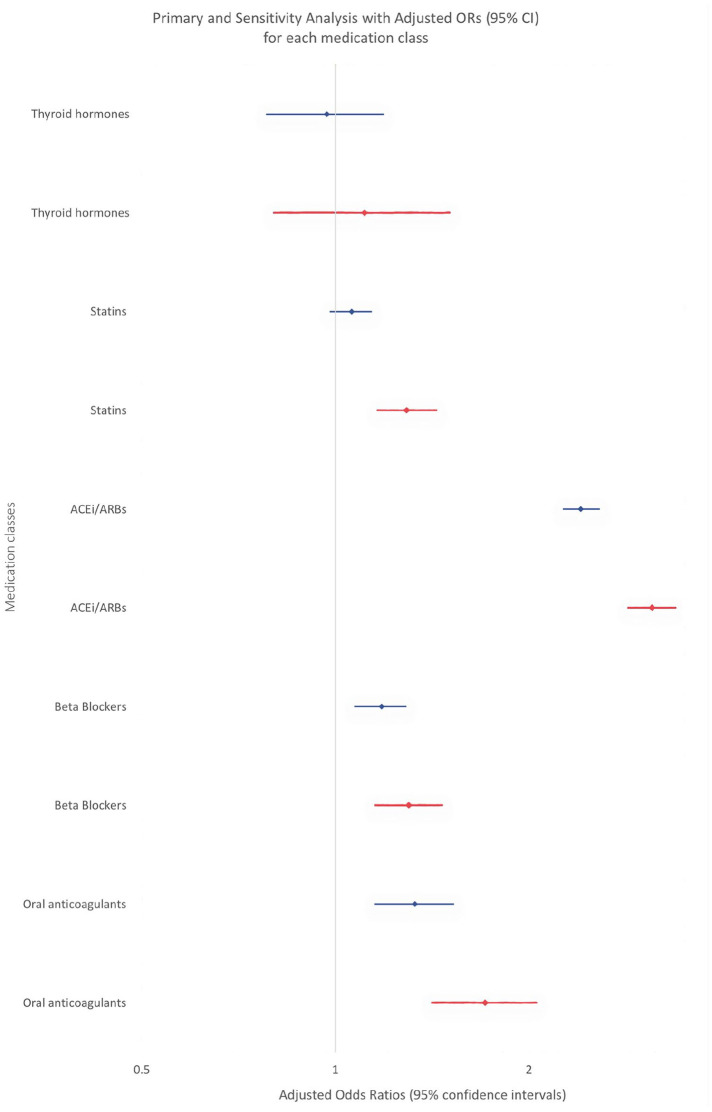
Forest plot for multivariable regression of the association of critical care and chronic medication discontinuation for each medication class in the primary and sensitivity analysis (*blue*: primary, *red*: sensitivity).

In sensitivity analyses, after excluding patients who died or were readmitted within 90 days of index hospital discharge, there was no appreciable change in discontinuation adjusted ORs for thyroid hormones, ACEi/ARBs, beta-blockers, and oral anticoagulants (Table 3 and Figure 3). For statins, critical care was associated with discontinuation (adjusted OR 1.29, 95%CI: 1.16–1.44).

**Table 3.** Sensitivity analysis for the association of critical care and discontinuation of medication classes, excluding patients who died or were readmitted within 90 days of index hospital discharge.

Medication class	Number of discontinuers/chronic critical care users (%)	Number of discontinuers/chronic non-critical care users (%)	Unadjusted OR (95% CI, *p*-value)	Adjusted^a^ OR (95% CI, *p*-value)
Thyroid hormones	50/896 (5.6)	538/10,040 (5.4)	1.04 (0.77–1.39, *p* = 0.778)	1.11 (0.80–1.51, *p* = 0.506)
Statins	491/4480 (11.0)	3035/32,507 (9.3)	1.20 (1.08–1.32, *p* = 0.001)	1.29 (1.16–1.44, *p* < 0.001)
ACEi/ARBs	1105/3818 (28.9)	3814/29,652 (12.9)	2.76 (2.55–2.98, *p* < 0.001)	3.11 (2.85–3.39, *p* < 0.001)
Beta-blocker	412/2814 (14.6)	2662/19,427 (13.7)	1.08 (0.96–1.21, *p* = 0.178)	1.30 (1.15–1.47, *p* < 0.001)
Oral anticoagulant	189/926 (20.4)	826/6085 (13.6)	1.63 (1.37–11.94, *p* < 0.001)	1.71 (1.41–12.06, *p* < 0.001)

OR: odds ratio; CI: confidence interval; ACEi: angiotensin-converting enzyme inhibitor; ARB: angiotensin receptor blocker.

a Adjusted models were adjusted for sex, age group, socioeconomic status, ethnicity, main condition at hospitalisation and comorbidity category.

#### Risk factors for medication discontinuation after index hospital discharge among critical care survivors

**Figure 4.**
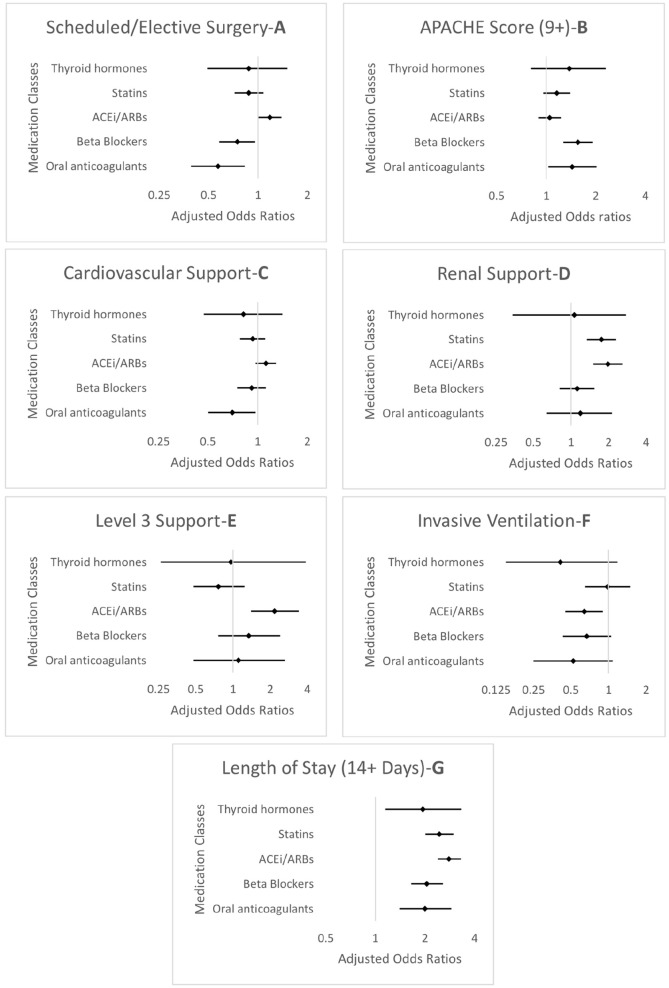
Forest plots (a–g) for multivariable regression models of the association between potential risk factors and chronic medication discontinuation among critical care survivors. Models were adjusted for sex, age group, SIMD, ethnicity, main condition, and index morbidity category (as for primary analysis), as well as surgery, APACHE II score, cardiovascular support, renal support, ventilatory support, highest level of support, and length of hospital stay.

In secondary analyses conducted to identify patient factors associated with medication discontinuation among critical care survivors, a length of stay of 14 or more days was the only factor associated with medication discontinuation across all medication classes, with adjusted ORs ranging from 1.27 (95%CI: 1.03–1.56) for statins to 2.80 (95%CI: 2.39–3.30) for ACEi/ARBs. For statins, renal support was associated with increased risk of discontinuation. For ACEi/ARBs, renal support, emergency surgery, elective surgery, and level 3 support were associated with increased risk of discontinuation while invasive ventilation was associated with reduced risk of discontinuation. For beta-blockers, an APACHE II score ⩾9 was associated with increased risk of discontinuation, while elective surgery was associated with reduced risk. For oral anticoagulants, an APACHE II score ⩾9 was associated with increased risk of discontinuation, while cardiovascular support and elective surgery were associated with reduced risk. APACHE II score, hospital length of stay, level 3 support, elective surgery, and organ support variables were plotted as forest plots (Figure 4). The full data for the medication classes are reported in Supplemental Table S7.

## Discussion

This study found an increased risk of discontinuation of ACEi/ARBs, beta blockers, and oral anticoagulants in the 90 days after hospital discharge in critical care compared with non-critical care hospitalised patients in Lothian, Scotland. Discontinuation of ACEi/ARBs was more than two-fold higher in critical care compared to non-critical care hospitalised survivors. There was no association observed between critical care admission and discontinuation of thyroid hormones or statins after hospital discharge. When we restricted our study population to critical care survivors, length of hospital stay ⩾14 days was the only factor associated with discontinuation across all studied medication classes.

Patients who experience a critical illness often have greater clinical complexity, a greater number of a care transitions, and greater ongoing healthcare needs after discharge from hospital.^4^ System performance around medication safety for critical care survivors transferring to a hospital ward is shaped by a complex interaction of care team, tasks, tools and technologies, organisational, physical environment, and external environmental factors.^17^ Perhaps this is best demonstrated by the association with reduced discontinuation of beta-blockers and oral anticoagulants after elective procedures, where patients have a more defined care process, care team involvement, and recovery pathway. In response, several interventions have been evaluated to improve medication continuity and safety in critical care survivors, including education of staff, medication review, guidelines, electronic transfer/handover tool or letter, and medicines reconciliation.^24^ Although multicomponent interventions based on staff education and guidelines are effective in reducing the risk of potentially inappropriate acute medication continuation,^24^ re-introduction of clinically important chronic medication may be of a higher clinical risk and a more complex problem to address.^16^

There was an increased risk of discontinuation of ACEi/ARBs in critical care survivors associated with both elective and emergency care pathways. ACEi/ARBs are routinely discontinued in both elective patients undergoing major surgical procedures and, in those emergency critical care patients with hypotension, acute kidney injury, or shock states. Clinical practice guidance supports discontinuation of ACEi/ARBs in these scenarios based on concerns about the hypotensive and potentially nephrotoxic effects.^25^ In patients recovering from an acute kidney injury, there is often a reluctance to restart ACEi/ARBs pending confirmation of full recovery to baseline kidney function. This recovery may be protracted, traversing the critical care to hospital ward, or community care interfaces. In turn this increases the requirement for, and complexity of, medication review and increases the potential for unintentional discontinuation.

Oral anticoagulants are often discontinued prior to elective procedures or on emergency critical care admission. They are high-risk medicines for medication errors and hospitals have well developed procedures for bleeding risk assessment, prophylactic venous thromboembolism prevention, and bridging anticoagulation treatment.^26^ The study’s results were largely consistent with previous research^27^ despite being the first to include all types of oral anticoagulants. Associated discontinuation risk was increased in more severely ill patients and those with longer hospital stays, but reduced in more predictable, elective cases.

The absence of observed inter-group differences in discontinuation rates for thyroid hormones and statins may reflect the more limited contraindications to these medication classes in the critically ill and responsiveness to routine delivery of medicines reconciliation.^16,20,28^

Increased length of hospital stay was associated with greater risk of medicines discontinuation in critical care survivors. This may reflect patient complexity and a more protracted recovery. In a recent systematic review of medication-related problems in critical care survivors, Short et al.^7^ found that length of stay was identified as a common risk factor for errors in medication. However, the risks were primarily related to continuation of potentially inappropriate medication. Previously, a three-center retrospective chart review of chronic medication discontinuation after an intensive care episode reported no association with length of stay.^14^ However, this study included only 834 patients and may have been underpowered to identify an association with length of stay and did not specifically analyse a more prolonged stay interval as we have done. In a later large population cohort study by the same group, critical care length of stay was not analysed as a factor that may have affected medication continuity.^10,29^

Our study had several strengths. It is among the first to investigate patient factors associated with the discontinuation of chronic medications among critical care survivors. In addition, our study had very few exclusions, and all exclusions that were present were objective and accounted for, allowing high internal validity. Furthermore, the large cohort sizes allow reasonable precision of estimates to be reported, reducing the risk of type II error. Finally, our 90 day evaluation period encompassed the usual period a patient may potentially be reviewed in a critical care follow up clinic^28^ with potential for medication changes, including recommencing chronic medication.^30^

There are also some limitations to this study. Despite the wealth of information our datasets contained, the major limitation is the lack of in-hospital medication and clinical decision-making details. Without this, we were unable to discern the appropriateness (either intentional or unintentional) or hospital unit (critical care vs general ward) of discontinuation. However, our additional analysis finding that 6.5% of patients on multiple medication classes studied had all of them discontinued may suggest unintentional discontinuation was not identified by existing hospital-wide medication safety systems. While our data did not provide detail of discharge location (e.g. patients discharged to their home vs care facilities), in Scotland, patients in care facilities do receive community prescriptions which would have been captured in the PIS data leveraged in our study. Another limitation is that patients had to have attended the emergency department during the study period to be included in our study, reducing the external validity to a “general” critical care population. Finally, medicine dosages and co-prescriptions were not assessed in our study. Polypharmacy could be a potential confounder and an independent factor for discontinuation, while changes in medicine dosage that may have resulted from the critical care admission may also have implications for potential clinical effectiveness and tolerability.

Further research is required to understand the reasons for increased risks of discontinuation of clinically important chronic medication in critical care survivors, in particular focussing on whether discontinuations are clinically indicated or a symptom of suboptimal systems in multiple transitions of care. More evidence of the impact of unintentional discontinuation of important chronic medication is also required to provide motivation and support for the systems and behavioural changes required to effectively mitigate unintentional discontinuation.^17^ Finally, further research on intervention packages to improve medication safety, including unintentional medication discontinuation, after a critical care patient episode are required.

## Conclusion

We found an association between critical care and the discontinuation of ACEi/ARBs, beta-blockers, and oral anticoagulants, but not thyroid hormone or statins. Hospital length of stay was the only consistent patient factor associated with discontinuation across all medication classes studied. This highlights the importance of further research to understand the impact of high-risk medication discontinuation on outcomes of critical care survivors and effective interventions to mitigate these.

## Supplemental Material

sj-docx-1-inc-10.1177_17511437241230260 – Supplemental material for Association between critical care admission and chronic medication discontinuation post-hospital discharge: A retrospective cohort study Supplemental material, sj-docx-1-inc-10.1177_17511437241230260 for Association between critical care admission and chronic medication discontinuation post-hospital discharge: A retrospective cohort study by Charvi Kanodia, Richard S Bourne, Elizabeth T Mansi and Nazir I Lone in Journal of the Intensive Care Society
