# Supplementary material for: Association between critical care admission and chronic medication discontinuation post-hospital discharge: A retrospective cohort study
Source: J Intensive Care Soc. 2024 Mar 1;25(3):255–65. doi: 10.1177/17511437241230260 (PMC11366179; doi:10.1177/17511437241230260)
Supplement: sj-docx-1-inc-10.1177_17511437241230260 – Supplemental material for Association between critical care admission and chronic medication discontinuation post-hospital discharge: A retrospective cohort study [file sj-docx-1-inc-10.1177_17511437241230260.docx]

**Supplementary Tables**

Association Between Critical Care Admission and Chronic Medication Discontinuation Post-Hospital Discharge: a Retrospective Cohort Study

Charvi Kanodia^1^, Richard S Bourne^2,3^, Elizabeth T Mansi^4^, Nazir I Lone^1,4^

^1^ Edinburgh Medical School, University of Edinburgh, UK

^2^ Departments of Pharmacy and Critical Care, Sheffield Teaching Hospitals NHS Foundation Trust, Sheffield, UK

^3^ Division of Pharmacy and Optometry, School of Health Sciences, Faculty of Biology, Medicine and Health, The University of Manchester, Manchester, UK

^4^ Usher Institute, University of Edinburgh, UK

Table of Contents

[**Table S1** Characteristics of adults hospitalised in Lothian who survived to hospital discharge (2012-2019) and were chronic *thyroid hormone* users prior to hospitalisation. 2](#_Toc142999448)

[**Table S2** Characteristics of adults hospitalised in Lothian who survived to hospital discharge (2012-2019) and were chronic *statin* users prior to hospitalisation. 3](#_Toc142999449)

[**Table S3** Characteristics of adults hospitalised in Lothian who survived to hospital discharge (2012-2019) and were chronic *ACEi or ARB* users prior to hospitalisation. 4](#_Toc142999450)

[**Table S4** Characteristics of adults hospitalised in Lothian who survived to hospital discharge (2012-2019) and were chronic *beta blockers* users prior to hospitalisation. 5](#_Toc142999451)

[**Table S5** Characteristics of adults hospitalised in Lothian who survived to hospital discharge (2012-2019) and were chronic *oral anticoagulant* users prior to hospitalisation. 6](#_Toc142999452)

[**Table S6** Multivariable model of the association between critical care and chronic medicine discontinuation, by specified medication class 7](#_Toc142999453)

[**Table S7** Multivariable model of the association between patient characteristics and chronic medicine discontinuation, by specified medication class (critical care survivors only) 9](#_Toc142999454)

### **Table S1** Characteristics of adults hospitalised in Lothian who survived to hospital discharge (2012-2019) and were chronic *thyroid hormone* users prior to hospitalisation.

| **Characteristics** | | **Critical care**  **n=1475 (%)** | **Non-critical care n=12,743 (%)** |
| --- | --- | --- | --- |
| Sex | Male | 386 (26.2) | 2135 (16.8) |
|  | Female | 1089 (73.8) | 10,608 (83.2) |
| Age | Median (IQR) | 71.0 (60.0 to 78.0) | 71.0 (57.0 to 81.0) |
| Age group | 18-29 | 18 (1.2) | 242 (1.9) |
|  | 30-39 | 34 (2.3) | 541 (4.2) |
|  | 40-49 | 112 (7.6) | 1093 (8.6) |
|  | 50-59 | 204 (13.8) | 1894 (14.9) |
|  | 60-69 | 331 (22.4) | 2260 (17.7) |
|  | 70-79 | 490 (33.2) | 2887 (22.7) |
|  | 80+ | 286 (19.4) | 3826 (30.0) |
| Socioeconomic status (SIMD quartile) | 5 (least deprived) | 375 (25.4) | 3327 (26.1) |
|  | 4 | 245 (16.6) | 2263 (17.8) |
|  | 3 | 267 (18.1) | 2158 (16.9) |
|  | 2 | 370 (25.1) | 3170 (24.9) |
|  | 1 (most deprived) | 210 (14.2) | 1728 (13.6) |
|  | Missing | <10 (0.5) | 97 (0.8) |
| Ethnicity | White | 1296 (87.9) | 10,798 (84.7) |
|  | Other | 11 (0.7) | 209 (1.6) |
|  | Unknown / Refused | 168 (11.4) | 1736 (13.6) |
| Main condition at hospitalisation | Digestive | 252 (17.1) | 1079 (8.5) |
|  | Circulatory | 304 (20.6) | 1221 (9.6) |
|  | Respiratory | 124 (8.4) | 1087 (8.5) |
|  | Abnormal Findings* | 47 (3.2) | 1774 (13.9) |
|  | Neoplasms | 278 (18.8) | 632 (5.0) |
|  | Injury | 158 (10.7) | 1556 (12.2) |
|  | Other | 312 (21.2) | 5394 (42.3) |
| Index morbidity count | none | 493 (33.4) | 7113 (55.8) |
|  | 1 | 250 (16.9) | 1899 (14.9) |
|  | 2 | 227 (15.4) | 1459 (11.4) |
|  | 3 or more | 505 (34.2) | 2272 (17.8) |
| Length of hospital stay (days) | Median (IQR) | 10.0 (6.0 to 21.0) | 1.0 (0.0 to 5.0) |
| Year discharged | 2012 | 162 (11.0) | 1585 (12.4) |
|  | 2013 | 179 (12.1) | 1551 (12.2) |
|  | 2014 | 192 (13.0) | 1582 (12.4) |
|  | 2015 | 201 (13.6) | 1549 (12.2) |
|  | 2016 | 188 (12.7) | 1541 (12.1) |
|  | 2017 | 198 (13.4) | 1496 (11.7) |
|  | 2018 | 187 (12.7) | 1597 (12.5) |
|  | 2019-2020 | 168 (11.4) | 1842 (14.4) |
| Hospital readmission within 90 days of hospital discharge |  | 551 (37.4) | 2369 (18.6) |
| Died within 90 days of hospital discharge |  | 73 (4.9) | 651 (5.1) |

*IQR:* interquartile range*; SIMD:* Scottish Index of Multiple Deprivation

**Abnormal findings:* International Classification of Diseases-10 codes R00-R99 (“Symptoms, signs and abnormal clinical and laboratory findings, not elsewhere classified”).

### **Table S2** Characteristics of adults hospitalised in Lothian who survived to hospital discharge (2012-2019) and were chronic *statin* users prior to hospitalisation.

| **Characteristics** | | **Critical care**  **n=7124 (%)** | **Non-critical care n=41,626 (%)** |
| --- | --- | --- | --- |
| Sex | Male | 4365 (61.3) | 21,387 (51.4) |
|  | Female | 2759 (38.7) | 20,239 (48.6) |
| Age | Median (IQR) | 70.0 (63.0 to 77.0) | 73.0 (64.0 to 81.0) |
| Age group | 18-39 | 41(0.6) | 251(0.6) |
|  | 40-49 | 281 (3.9) | 1535 (3.7) |
|  | 50-59 | 941 (13.2) | 5173 (12.4) |
|  | 60-69 | 2056 (28.9) | 9640 (23.2) |
|  | 70-79 | 2560 (35.9) | 13,202 (31.7) |
|  | 80+ | 1245 (17.5) | 11,825 (28.4) |
| Socioeconomic status (SIMD quartile) | 5 (least deprived) | 1733 (24.3) | 10,379 (24.9) |
|  | 4 | 1098 (15.4) | 7008 (16.8) |
|  | 3 | 1251 (17.6) | 7462 (17.9) |
|  | 2 | 1903 (26.7) | 10,549 (25.3) |
|  | 1 (most deprived) | 1109 (15.6) | 6010 (14.4) |
|  | Missing | 30 (0.4) | 218 (0.5) |
| Ethnicity | White | 6100 (85.6) | 35,006 (84.1) |
|  | Other | 112 (1.5) | 705 (1.7) |
|  | Unknown / Refused | 912 (12.8) | 5915 (14.2) |
| Main condition at hospitalisation | Digestive | 1018 (14.3) | 3199 (7.7) |
|  | Circulatory | 2108 (29.6) | 5941 (14.3) |
|  | Respiratory | 562 (7.9) | 3381 (8.1) |
|  | Abnormal Findings* | 213 (3.0) | 5889 (14.1) |
|  | Neoplasms | 1272 (17.9) | 2182 (5.2) |
|  | Injury | 701 (9.8) | 4389 (10.5) |
|  | Other | 1250 (17.5) | 16,645 (40.0) |
| Index morbidity count | none | 2358 (33.1) | 21,128 (50.8) |
|  | 1 | 1296 (18.2) | 7322 (17.6) |
|  | 2 | 1213 (17.0) | 5751 (13.8) |
|  | 3 or more | 2257 (31.7) | 7425 (17.8) |
| Length of hospital stay (days) | Median (IQR) | 10.0 (6.0 to 20.0) | 1.0 (0.0 to 5.0) |
| Year discharged | 2012 | 842 (11.8) | 5098 (12.2) |
|  | 2013 | 932 (13.1) | 5033 (12.1) |
|  | 2014 | 970 (13.6) | 5102 (12.3) |
|  | 2015 | 870 (12.2) | 5050 (12.1) |
|  | 2016 | 905 (12.7) | 4786 (11.5) |
|  | 2017 | 862 (12.1) | 5037 (12.1) |
|  | 2018 | 866 (12.2) | 5190 (12.5) |
|  | 2019 | 845 (11.9) | 6225 (15.0) |
|  | 2020 | 32 (0.4) | 105 (0.3) |
| Hospital readmission within 90 days of hospital discharge |  | 2537 (35.6) | 8163 (19.6) |
| Died within 90 days of hospital discharge |  | 304 (4.3) | 2084 (5.0) |

*IQR:* interquartile range*; SIMD:* Scottish Index of Multiple Deprivation

**Abnormal findings:* International Classification of Diseases-10 codes R00-R99 (“Symptoms, signs and abnormal clinical and laboratory findings, not elsewhere classified”).

### **Table S3** Characteristics of adults hospitalised in Lothian who survived to hospital discharge (2012-2019) and were chronic *ACEi or ARB* users prior to hospitalisation.

| **Characteristics** | | **Critical care**  **n=5974 (%)** | **Non-critical care n=37,231(%)** |
| --- | --- | --- | --- |
| Sex | Male | 3593 (60.1) | 18430 (49.5) |
|  | Female | 2381 (39.9) | 18801 (50.5) |
| Age | Median (IQR) | 70.0 (61.0 to 77.0) | 72.0 (61.0 to 80.0) |
| Age group | 18-29 | 28 (0.5) | 78 (0.2) |
|  | 30-39 | 57 (1.0) | 411 (1.1) |
|  | 40-49 | 273 (4.6) | 2077 (5.6) |
|  | 50-59 | 865 (14.5) | 5476 (14.7) |
|  | 60-69 | 1685 (28.2) | 8448 (22.7) |
|  | 70-79 | 2028 (33.9) | 10597 (28.5) |
|  | 80+ | 1038 (17.4) | 10144 (27.2) |
| Socioeconomic status (SIMD quartile) | 5 (least deprived) | 1526 (25.5) | 9903 (26.6) |
|  | 4 | 950 (15.9) | 6410 (17.2) |
|  | 3 | 1073 (18.0) | 6683 (18.0) |
|  | 2 | 1538 (25.7) | 9170 (24.6) |
|  | 1 (most deprived) | 856 (14.3) | 4850 (13.0) |
|  | Missing | 31 (0.5) | 215 (0.6) |
| Ethnicity | White | 5098 (85.3) | 31078 (83.5) |
|  | Other | 93 (1.6) | 608 (1.6) |
|  | Unknown / Refused | 783 (13.1) | 5545 (14.9) |
| Main condition at hospitalisation | Digestive | 953 (16.0) | 3082 (8.3) |
|  | Circulatory | 1671 (28.0) | 5351 (14.4) |
|  | Respiratory | 484 (8.1) | 2614 (7.0) |
|  | Abnormal Findings* | 172 (2.9) | 5001 (13.4) |
|  | Neoplasms | 1098 (18.4) | 1958 (5.3) |
|  | Injury | 564 (9.4) | 3968 (10.7) |
|  | Other | 1032 (17.3) | 15257 (41.0) |
| Index morbidity count | none | 2200 (36.8) | 20121 (54.0) |
|  | 1 | 1026 (17.2) | 6157 (16.5) |
|  | 2 | 973 (16.3) | 4942 (13.3) |
|  | 3 or more | 1775 (29.7) | 6011 (16.1) |
| Length of hospital stay (days) | Median (IQR) | 11.0 (6.0 to 20.8) | 1.0 (0.0 to 4.0) |
| Year discharged | 2012 | 744 (12.5) | 4512 (12.1) |
|  | 2013 | 792 (13.3) | 4482 (12.0) |
|  | 2014 | 810 (13.6) | 4621 (12.4) |
|  | 2015 | 787 (13.2) | 4560 (12.2) |
|  | 2016 | 751 (12.6) | 4294 (11.5) |
|  | 2017 | 723 (12.1) | 4513 (12.1) |
|  | 2018 | 685 (11.5) | 4754 (12.8) |
|  | 2019 | 665 (11.1) | 5408 (14.5) |
|  | 2020 | 17 (0.3) | 87 (0.2) |
| Hospital readmission within 90 days of hospital discharge |  | 2084 (34.9) | 6829 (18.3) |
| Died within 90 days of hospital discharge |  | 225 (3.8) | 1599 (4.3) |

*IQR:* interquartile range*; SIMD:* Scottish Index of Multiple Deprivation; *ACEi:* angiotensin-converting enzyme inhibitor; *ARB:* angiotensin receptor blocker

**Abnormal findings:* International Classification of Diseases-10 codes R00-R99 (“Symptoms, signs and abnormal clinical and laboratory findings, not elsewhere classified”).

### **Table S4** Characteristics of adults hospitalised in Lothian who survived to hospital discharge (2012-2019) and were chronic *beta blockers* users prior to hospitalisation.

| **Characteristics** | | **Critical care**  **n=4454 (%)** | **Non-critical care n=25,283 (%)** |
| --- | --- | --- | --- |
| Sex | Male | 2689 (60.4) | 11984 (47.4) |
|  | Female | 1765 (39.6) | 13299 (52.6) |
| Age | Median (IQR) | 70.0 (61.0 to 77.0) | 73.0 (62.0 to 81.0) |
| Age group | 18-29 | 49 (1.1) | 507 (2.0) |
|  | 30-39 | 100 (2.2) | 740 (2.9) |
|  | 40-49 | 258 (5.8) | 1356 (5.4) |
|  | 50-59 | 611 (13.7) | 2932 (11.6) |
|  | 60-69 | 1182 (26.5) | 4984 (19.7) |
|  | 70-79 | 1453 (32.6) | 6994 (27.7) |
|  | 80+ | 801 (18.0) | 7770 (30.7) |
| Socioeconomic status (SIMD quartile) | 5 (least deprived) | 1101 (24.7) | 6441 (25.5) |
|  | 4 | 691 (15.5) | 4297 (17.0) |
|  | 3 | 773 (17.4) | 4527 (17.9) |
|  | 2 | 1198 (26.9) | 6378 (25.2) |
|  | 1 (most deprived) | 672 (15.1) | 3478 (13.8) |
|  | Missing | 19 (0.4) | 162 (0.6) |
| Ethnicity | White | 3795 (85.2) | 21317 (84.3) |
|  | Other | 78 (1.8) | 357 (1.4) |
|  | Unknown / Refused | 581 (13.0) | 3609 (14.3) |
| Main condition at hospitalisation | Digestive | 646 (14.5) | 1942 (7.7) |
|  | Circulatory | 1398 (31.4) | 4490 (17.8) |
|  | Respiratory | 277 (6.2) | 1649 (6.5) |
|  | Abnormal Findings* | 138 (3.1) | 3788 (15.0) |
|  | Neoplasms | 693 (15.6) | 1250 (4.9) |
|  | Injury | 470 (10.6) | 2698 (10.7) |
|  | Other | 832 (18.7) | 9466 (37.4) |
| Index morbidity count | none | 1345 (30.2) | 12164 (48.1) |
|  | 1 | 809 (18.2) | 4587 (18.1) |
|  | 2 | 710 (15.9) | 3507 (13.9) |
|  | 3 or more | 1590 (35.7) | 5025 (19.9) |
| Length of hospital stay (days) | Median (IQR) | 10.0 (6.0 to 20.0) | 1.0 (0.0 to 5.0) |
| Year discharged | 2012 | 505 (11.3) | 3250 (12.9) |
|  | 2013 | 544 (12.2) | 3080 (12.2) |
|  | 2014 | 625 (14.0) | 3139 (12.4) |
|  | 2015 | 560 (12.6) | 3080 (12.2) |
|  | 2016 | 546 (12.3) | 2966 (11.7) |
|  | 2017 | 589 (13.2) | 3004 (11.9) |
|  | 2018 | 537 (12.1) | 3138 (12.4) |
|  | 2019 | 526 (11.8) | 3564 (14.1) |
|  | 2020 | 22 (0.5) | 62 (0.2) |
| Hospital readmission within 90 days of hospital discharge |  | 1576 (35.4) | 5197 (20.6) |
| Died within 90 days of hospital discharge |  | 192 (4.3) | 1392 (5.5) |

*IQR:* interquartile range*; SIMD:* Scottish Index of Multiple Deprivation

**Abnormal findings:* International Classification of Diseases-10 codes R00-R99 (“Symptoms, signs and abnormal clinical and laboratory findings, not elsewhere classified”).

### **Table S5** Characteristics of adults hospitalised in Lothian who survived to hospital discharge (2012-2019) and were chronic *oral anticoagulant* users prior to hospitalisation.

| **Characteristics** | | **Critical care**  **n=1461 (%)** | **Non-critical care n=8471 (%)** |
| --- | --- | --- | --- |
| Sex | Male | 885 (60.6) | 4468 (52.7) |
|  | Female | 576 (39.4) | 4003 (47.3) |
| Age | Median (IQR) | 73.0 (64.0 to 79.0) | 78.0 (69.0 to 84.0) |
| Age group | 18-39 | 35 (2.4) | 129 (1.6) |
|  | 40-49 | 53 (3.6) | 269 (3.2) |
|  | 50-59 | 160 (11.0) | 510 (6.0) |
|  | 60-69 | 320 (21.9) | 1277 (15.1) |
|  | 70-79 | 529 (36.2) | 2659 (31.4) |
|  | 80+ | 364 (24.9) | 3627 (42.8) |
| Socioeconomic status (SIMD quartile) | 5 (least deprived) | 423 (29.0) | 2588 (30.6) |
|  | 4 | 262 (17.9) | 1508 (17.8) |
|  | 3 | 252 (17.2) | 1469 (17.3) |
|  | 2 | 331 (22.7) | 1902 (22.5) |
|  | 1 (most deprived) | 183 (12.5) | 957 (11.3) |
|  | Missing | 10 (0.7) | 47 (0.6) |
| Ethnicity | White | 1282 (87.7) | 7325 (86.5) |
|  | Other | 19 (1.3) | 51 (0.6) |
|  | Unknown / Refused | 160 (11.0) | 1095 (12.9) |
| Main condition at hospitalisation | Digestive | 242 (16.6) | 549 (6.5) |
|  | Circulatory | 420 (28.7) | 1639 (19.3) |
|  | Respiratory | 115 (7.9) | 732 (8.6) |
|  | Abnormal Findings* | 55 (3.8) | 1261 (14.9) |
|  | Neoplasms | 194 (13.3) | 394 (4.7) |
|  | Injury | 187 (12.8) | 960 (11.3) |
|  | Other | 248 (17.0) | 2936 (34.7) |
| Index morbidity count | none | 261 (17.9) | 2394 (28.3) |
|  | 1 | 228 (15.6) | 1588 (18.7) |
|  | 2 | 257 (17.6) | 1503 (17.7) |
|  | 3 or more | 715 (48.9) | 2986 (35.2) |
| Length of hospital stay (days) | Median (IQR) | 12.0 (7.0 to 23.0) | 2.0 (0.0 to 6.5) |
| Year discharged | 2012 | 119 (8.1) | 876 (10.3) |
|  | 2013 | 145 (9.9) | 860 (10.2) |
|  | 2014 | 166 (11.4) | 908 (10.7) |
|  | 2015 | 200 (13.7) | 948 (11.2) |
|  | 2016 | 197 (13.5) | 1011 (11.9) |
|  | 2017 | 219 (15.0) | 1079 (12.7) |
|  | 2018 | 178 (12.2) | 1212 (14.3) |
|  | 2019-2020 | 237 (16.2) | 1577(18.6) |
| Hospital readmission within 90 days of hospital discharge |  | 512 (35.0) | 2139 (25.3) |
| Died within 90 days of hospital discharge |  | 71 (4.9) | 582 (6.9) |

*IQR:* interquartile range*; SIMD:* Scottish Index of Multiple Deprivation

**Abnormal findings:* International Classification of Diseases-10 codes R00-R99 (“Symptoms, signs and abnormal clinical and laboratory findings, not elsewhere classified”).

**Table S6** Multivariable model of the association between critical care and chronic medicine discontinuation, by specified medication class.

|  | | **Thyroid hormones (%)** | **Adjusted OR**  **(95% CI,**  **p-value)** | **Statins (%)** | **Adjusted OR**  **(95% CI,**  **p-value)** | **ACEi/ARBs (%)** | **Adjusted OR**  **(95% CI,**  **p-value)** | **Beta blockers**  **(%)** | **Adjusted OR**  **(95% CI,**  **p-value)** | **Oral Anticoagulants (%)** | **Adjusted OR**  **(95% CI,**  **p-value)** |
| --- | --- | --- | --- | --- | --- | --- | --- | --- | --- | --- | --- |
| **Type of hospitalisation** | Non-critical care | 969 (7.6) | Reference | 5446 (13.1) | Reference | 6403 (17.2) | Reference | 4096 (16.2) | Reference | 1578 (18.6) | Reference |
|  | Critical care | 116 (7.9) | 0.96  (0.77-1.18, p=0.700) | 1030 (14.5) | 1.05  (0.97-1.13, p=0.243) | 1986 (33.2) | 2.39  (2.24-2.56, p<0.001) | 784 (17.6) | 1.16  (1.06-1.27, p=0.001) | 353 (24.2) | 1.33  (1.15-1.52, p<0.001) |
| **Sex** | Male | 207 (8.2) | Reference | 3232 (12.6) | Reference | 4152 (18.9) | Reference | 2254 (15.4) | Reference | 1041 (19.4) | Reference |
|  | Female | 878 (7.5) | 0.93  (0.79-1.09, p=0.371) | 3244 (14.1) | 1.07  (1.02-1.13, p=0.011) | 4237 (20.0) | 1.02  (0.97-1.07, p=0.436) | 2626 (17.4) | 1.07  (1.01-1.14, p=0.034) | 890 (19.4) | 0.96  (0.86-1.06, p=0.399) |
| **Age group** | 18-29 | 31 (11.9) | Reference | <10 | Reference | 26 (24.5) | Reference | 229 (41.2) | Reference | 13 (28.3) | Reference |
|  | 30-39 | 56 (9.7) | 0.85  (0.53-1.39, p=0.511) | 41 (15.8) | 0.70  (0.30-1.85, p=0.435) | 79 (16.9) | 0.70  (0.42-1.18, p=0.171) | 258 (30.7) | 0.64  (0.51-0.81, p<0.001) | 35 (29.7) | 1.02  (0.49-2.24, p=0.954) |
|  | 40-49 | 88 (7.3) | 0.60  (0.39-0.95, p=0.025) | 204 (11.2) | 0.46  (0.21-1.16, p=0.072) | 283 (12.0) | 0.47  (0.29-0.76, p=0.002) | 296 (18.3) | 0.32  (0.26-0.40, p<0.001) | 67 (20.8) | 0.63  (0.32-1.30, p=0.191) |
|  | 50-59 | 133 (6.3) | 0.50  (0.33-0.78, p=0.001) | 623 (10.2) | 0.40  (0.18-1.01, p=0.033) | 823 (13.0) | 0.49  (0.32-0.80, p=0.003) | 508 (14.3) | 0.24  (0.20-0.30, p<0.001) | 135 (20.1) | 0.58  (0.30-1.17, p=0.110) |
|  | 60-69 | 148 (5.7) | 0.42  (0.28-0.66, p<0.001) | 1147 (9.8) | 0.36  (0.16-0.91, p=0.018) | 1519 (15.0) | 0.54  (0.35-0.87, p=0.009) | 749 (12.1) | 0.20  (0.16-0.24, p<0.001) | 260 (16.3) | 0.42  (0.22-0.85, p=0.011) |
|  | 70-79 | 217 (6.4) | 0.46  (0.31-0.71, p<0.001) | 1834 (11.6) | 0.43  (0.19-1.07, p=0.047) | 2388 (18.9) | 0.71  (0.45-1.14, p=0.144) | 1136 (13.4) | 0.22  (0.18-0.26, p<0.001) | 545 (17.1) | 0.45  (0.24-0.90, p=0.018) |
|  | 80+ | 412 (10.0) | 0.74  (0.50-1.13, p=0.145) | 2620 (20.0) | 0.81  (0.37-2.03, p=0.617) | 3271 (29.3) | 1.35  (0.86-2.17, p=0.204) | 1704 (19.9) | 0.35  (0.29-0.42, p<0.001) | 876 (21.9) | 0.64  (0.34-1.28, p=0.185) |
| **SIMD** | 5 | 281 (7.6) | Reference | 1604 (13.2) | Reference | 2268 (19.8) | Reference | 1234 (16.4) | Reference | 571 (19.0) | Reference |
|  | 4 | 198 (7.9) | 1.07  (0.89-1.30, p=0.465) | 1156 (14.3) | 1.12  (1.03-1.22, p=0.006) | 1444 (19.6) | 1.06  (0.98-1.14, p=0.164) | 805 (16.1) | 0.97  (0.88-1.08, p=0.603) | 368 (20.8) | 1.13  (0.97-1.31, p=0.107) |
|  | 3 | 178 (7.3) | 0.99  (0.81-1.20) | 1196 (13.7) | 1.10  (1.01-1.20) | 1468 (18.9) | 1.03  (0.96-1.12, p=0.375) | 897 (16.9) | 1.03  (0.94-1.14, p=0.527) | 328 (19.1) | 1.01  (0.87-1.18, p=0.872) |
|  | 2 | 261 (7.4) | 1.01  (0.84-1.21, p=0.920) | 1557 (12.5) | 0.99  (0.92-1.07, p=0.801) | 2066 (19.3) | 1.07  (0.99-1.14, p=0.071) | 1177 (15.5) | 0.95  (0.87-1.03, p=0.228) | 425 (19.0) | 0.99  (0.86-1.15, p=0.935) |
|  | 1 | 161 (8.3) | 1.12  (0.91-1.38, p=0.272) | 928 (13.0) | 1.07  (0.98-1.17, p=0.154) | 1083 (19.0) | 1.06  (0.98-1.16, p=0.157) | 726 (17.5) | 1.07  (0.96-1.18, p=0.233) | 229 (20.1) | 1.08  (0.91-1.29, p=0.381) |
| **Ethnicity** | White | 920 (7.6) | Reference | 5504 (13.4) | Reference | 7058 (19.5) | Reference | 4147 (16.5) | Reference | 1701 (19.8) | Reference |
|  | Asian | 14 (8.6) | 1.19  (0.65-2.01, p=0.552) | 67 (10.9) | 0.92  (0.70-1.18, p=0.531) | 82 (16.0) | 0.98  (0.76-1.24, p=0.859) | 44 (14.6) | 0.92  (0.65-1.26, p=0.597) | <10 | 0.89  (0.38-1.86, p=0.768) |
|  | Black | <10 | 1.66  (0.39-4.85, p=0.411) | 14 (14.7) | 1.37  (0.74-2.36, p=0.287) | 14 (15.4) | 1.02  (0.55-1.78, p=0.942) | <10 | 0.85  (0.37-1.72, p=0.677) | <10 | 0.57  (0.09-2.10, p=0.469) |
|  | Unknown / Refused | 146 (7.7) | 1.07  (0.88-1.28, p=0.486) | 874 (12.8) | 1.03  (0.95-1.11, p=0.459) | 1214 (19.2) | 1.06  (0.99-1.14, p=0.111) | 667 (15.9) | 0.99  (0.90-1.08, p=0.779) | 218 (17.4) | 0.89  (0.76-1.04, p=0.153) |
|  | Mixed / Other | <10 | 0.83  (0.13-2.77, p=0.795) | 17 (16.0) | 1.42  (0.81-2.35, p=0.192) | 21 (21.2) | 1.44  (0.85-2.34, p=0.151) | 14 (18.2) | 1.03  (0.54-1.83, p=0.930) | <10 | 0.60  (0.09-2.32, p=0.519) |
| **Main condition**  **at hospitalisation** | Digestive | 81 (6.1) | Reference | 502 (11.9) | Reference | 680 (16.9) | Reference | 387 (15.0) | Reference | 152 (19.2) | Reference |
|  | Circulatory | 118 (7.7) | 1.24  (0.93-1.68, p=0.150) | 889 (11.0) | 0.87  (0.77-0.98, p=0.022) | 1460 (20.8) | 1.18  (1.07-1.31, p=0.002) | 953 (16.2) | 1.20  (1.05-1.37, p=0.006) | 348 (16.9) | 0.88  (0.71-1.09, p=0.250) |
|  | Respiratory | 143 (11.8) | 1.91  (1.43-2.56, p<0.001) | 686 (17.4) | 1.40  (1.23-1.59, p<0.001) | 789 (25.5) | 1.59  (1.41-1.80, p<0.001) | 384 (19.9) | 1.45  (1.24-1.70, p<0.001) | 189 (22.3) | 1.22  (0.96-1.56, p=0.104) |
|  | Abnormal Findings* | 143 (7.9) | 1.19  (0.90-1.59, p=0.231) | 951 (15.6) | 1.26  (1.12-1.42, p<0.001) | 1127 (21.8) | 1.49  (1.33-1.66, p<0.001) | 773 (19.7) | 1.41  (1.23-1.62, p<0.001) | 271 (20.6) | 1.14  (0.91-1.43, p=0.259) |
|  | Neoplasms | 105 (11.5) | 2.04  (1.50-2.79, p<0.001) | 806 (23.3) | 2.16  (1.91-2.45, p<0.001) | 930 (30.4) | 1.77  (1.58-1.99, p<0.001) | 366 (18.8) | 1.48  (1.26-1.74, p<0.001) | 189 (32.1) | 2.09  (1.63-2.69, p<0.001) |
|  | Injury | 152 (8.9) | 1.40  (1.06-1.86, p=0.021) | 816 (16.0) | 1.28  (1.14-1.45, p<0.001) | 1045 (23.1) | 1.50  (1.34-1.67, p<0.001) | 685 (21.6) | 1.42  (1.23-1.63, p<0.001) | 264 (23.0) | 1.30  (1.03-1.63, p=0.025) |
|  | Other | 343 (6.0) | 0.96  (0.75-1.25, p=0.773) | 1826 (10.2) | 0.83  (0.74-0.92, p=0.001) | 2358 (14.5) | 0.96  (0.87-1.06, p=0.380) | 1332 (12.9) | 0.86  (0.76-0.98, p=0.019) | 518 (16.3) | 0.88  (0.72-1.08, p=0.212) |
| **Index morbidity count (5yr)** | none | 514 (6.8) | Reference | 2620 (11.2) | Reference | 3599 (16.1) | Reference | 2230 (16.5) | Reference | 460 (17.3) | Reference |
|  | 1 | 157 (7.3) | 0.98  (0.81-1.18, p=0.823) | 1058 (12.3) | 1.04  (0.96-1.12, p=0.338) | 1335 (18.6) | 1.09  (1.01-1.17, p=0.018) | 841 (15.6) | 0.92  (0.84-1.00, p=0.057) | 334 (18.4) | 1.06 (0.91-1.25, p=0.437) |
|  | 2 | 142 (8.4) | 1.14  (0.93-1.39, p=0.193) | 1033 (14.8) | 1.24  (1.14-1.34, p<0.001) | 1286 (21.7) | 1.24  (1.16-1.34, p<0.001) | 636 (15.1) | 0.89  (0.80-0.98, p=0.016) | 359 (20.4) | 1.18  (1.01-1.38, p=0.036) |
|  | 3 or more | 272 (9.8) | 1.32  (1.12-1.55, p=0.001) | 1765 (18.2) | 1.55  (1.45-1.66, p<0.001) | 2169 (27.9) | 1.59  (1.49-1.69, p<0.001) | 1173 (17.7) | 1.08  (0.99-1.17, p=0.077) | 778 (21.0) | 1.24 (1.08-1.41, p=0.002) |

*Note:* Cell values less than 10 are censored. **Abnormal findings:* International Classification of Diseases-10 codes R00-R99 (“Symptoms, signs and abnormal clinical and laboratory findings, not elsewhere classified”). *SIMD:* Scottish Index of Multiple Deprivation; *OR:* odds ratio; *CI:* confidence interval

**Table S7** Multivariable model of the association between patient characteristics and chronic medicine discontinuation, by specified medication class (critical care survivors only).

|  | | **Thyroid hormones (%)** | **Adjusted OR**  **(95% CI,**  **p-value)** | **Statins (%)** | **Adjusted OR**  **(95% CI,**  **p-value)** | **ACEi/ARBs (%)** | **Adjusted OR**  **(95% CI,**  **p-value)** | **Beta blockers**  **(%)** | **Adjusted OR**  **(95% CI,**  **p-value)** | **Oral Anticoagulants (%)** | **Adjusted OR**  **(95% CI,**  **p-value)** |
| --- | --- | --- | --- | --- | --- | --- | --- | --- | --- | --- | --- |
| **Sex** | Male | 29 (7.5) | Reference | 606 (13.9) | Reference | 1203 (33.5) | Reference | 447 (16.6) | Reference | 213 (24.1) | Reference |
|  | Female | 87 (8.0) | 1.12  (0.72-1.78, p=0.619) | 424 (15.4) | 1.12  (0.72-1.78, p=0.619) | 783 (32.9) | 0.98  (0.88-1.11, p=0.780) | 337 (19.1) | 1.12  (0.95-1.32, p=0.166) | 140 (24.3) | 1.00  (0.78-1.29, p=0.978) |
| **Age group** | 18-49 | 17 (10.4) | Reference | 45 (14.0) | Reference | 132 (36.9) | Reference | 93 (22.9) | Reference | 23 (26.1) | Reference |
|  | 50-74 | 53 (6.9) | 0.56  (0.31-1.05, p=0.059) | 600 (13.8) | 0.56  (0.31-1.05, p=0.059) | 1155 (31.9) | 0.79  (0.62-1.00, p=0.050) | 409 (16.2) | 0.77  (0.58-1.01, p=0.059) | 173 (23.5) | 0.85  (0.50-1.47, p=0.546) |
|  | 75 and over | 46 (8.5) | 0.62  (0.33-1.20, p=0.145) | 385 (15.6) | 0.62  (0.33-1.20, p=0.145) | 699 (35.0) | 0.85  (0.66-1.10, p=0.213) | 282 (18.6) | 0.82  (0.61-1.11, p=0.190) | 157 (24.6) | 0.77  (0.45-1.36, p=0.355) |
| **SIMD** | 5 | 30 (8.0) | Reference | 232 (13.4) | Reference | 518 (33.9) | Reference | 188 (17.1) | Reference | 99 (23.4) | Reference |
|  | 4 | 23 (9.4) | 1.19  (0.65-2.13, p=0.567) | 162 (14.8) | 1.19  (0.65-2.13, p=0.567) | 323 (34.0) | 0.99  (0.83-1.19, p=0.929) | 127 (18.4) | 1.07  (0.83-1.38, p=0.586) | 68 (26.0) | 1.12  (0.77-1.62, p=0.548) |
|  | 3 | 20 (7.5) | 0.87  (0.47-1.58, p=0.645) | 198 (15.8) | 0.87  (0.47-1.58, p=0.645) | 355 (33.1) | 0.96  (0.80-1.14, p=0.613) | 139 (18.0) | 1.02  (0.80-1.31, p=0.857) | 59 (23.4) | 0.96  (0.65-1.41, p=0.844) |
|  | 2 | 27 (7.3) | 0.88  (0.50-1.54, p=0.647) | 266 (14.0) | 0.88  (0.50-1.54, p=0.647) | 495 (32.2) | 0.92  (0.79-1.08, p=0.309) | 191 (15.9) | 0.87  (0.69-1.10, p=0.238) | 80 (24.2) | 0.97  (0.68-1.39, p=0.888) |
|  | 1 | 16 (7.6) | 0.94  (0.48-1.79, p=0.849) | 165 (14.9) | 0.94  (0.48-1.79, p=0.849) | 284 (33.2) | 0.93  (0.77-1.12, p=0.444) | 136 (20.2) | 1.13  (0.87-1.46, p=0.352) | 45 (24.6) | 1.05  (0.68-1.60, p=0.818) |
| **Ethnicity** | White | 99 (7.6) | Reference | 889 (14.6) | Reference | 1666 (32.7) | Reference | 661 (17.4) | Reference | 312 (24.3) | Reference |
|  | Non-White or Unknown | 17 (9.5) | 1.40  (0.77-2.42, p=0.243) | 141 (13.8) | 1.40  (0.77-2.42, p=0.243) | 320 (36.5) | 1.24  (1.06-1.45, p=0.008) | 123 (18.7) | 1.14  (0.91-1.42, p=0.249) | 41 (22.9) | 0.96  (0.65-1.41, p=0.857) |
| **Main condition at hospitalisation** | Digestive | 13 (5.2) | Reference | 156 (15.3) | Reference | 266 (27.9) | Reference | 125 (19.3) | Reference | 58 (24.0) | Reference |
|  | Circulatory | 24 (7.9) | 1.68  (0.81-3.61, p=0.171) | 218 (10.3) | 1.68  (0.81-3.61, p=0.171) | 584 (34.9) | 1.25  (1.02-1.53, p=0.032) | 200 (14.3) | 0.81  (0.62-1.07, p=0.139) | 85 (20.2) | 0.95  (0.62-1.47, p=0.833) |
|  | Respiratory | 13 (10.5) | 1.87  (0.78-4.45, p=0.157) | 90 (16.0) | 1.87  (0.78-4.45, p=0.157) | 176 (36.4) | 1.51  (1.17-1.95, p=0.001) | 61 (22.0) | 1.06  (0.73-1.51, p=0.765) | 25 (21.7) | 0.76  (0.42-1.33, p=0.345) |
|  | Abnormal Clin/Lab Findings | <10 | 0.30  (0.02-1.63, p=0.257) | 40 (18.8) | 0.30  (0.02-1.63, p=0.257) | 73 (42.4) | 1.85  (1.30-2.63, p=0.001) | 29 (21.0) | 1.00  (0.62-1.58, p=0.987) | 17 (30.9) | 1.29  (0.64-2.49, p=0.466) |
|  | Neoplasms | 27 (9.7) | 2.07  (1.03-4.36, p=0.047) | 200 (15.7) | 2.07  (1.03-4.36, p=0.047) | 287 (26.1) | 1.01  (0.82-1.25, p=0.895) | 92 (13.3) | 0.76  (0.56-1.03, p=0.078) | 49 (25.3) | 1.21  (0.77-1.92, p=0.410) |
|  | Injury | 16 (10.1) | 1.68  (0.76-3.77, p=0.198) | 116 (16.5) | 1.68  (0.76-3.77, p=0.198) | 201 (35.6) | 1.39  (1.09-1.76, p=0.007) | 124 (26.4) | 1.34  (1.00-1.81, p=0.050) | 58 (31.0) | 1.26  (0.80-1.98, p=0.310) |
|  | Other | 22 (7.1) | 1.16  (0.56-2.50, p=0.697) | 210 (16.8) | 1.16  (0.56-2.50, p=0.697) | 399 (38.7) | 1.50  (1.22-1.84, p<0.001) | 153 (18.4) | 0.89  (0.67-1.17, p=0.404) | 61 (24.6) | 0.86  (0.55-1.34, p=0.502) |
| **Index morbidity count (5yr)** | none | 30 (6.1) | Reference | 327 (13.9) | Reference | 667 (30.3) | Reference | 248 (18.4) | Reference | 65 (24.9) | Reference |
|  | 1 | 16 (6.4) | 1.06  (0.54-1.98, p=0.868) | 170 (13.1) | 1.06  (0.54-1.98, p=0.868) | 339 (33.0) | 1.23  (1.04-1.45, p=0.016) | 128 (15.8) | 0.99  (0.78-1.26, p=0.958) | 68 (29.8) | 1.28  (0.84-1.93, p=0.251) |
|  | 2 | 20 (8.8) | 1.55  (0.84-2.82, p=0.156) | 187 (15.4) | 1.55  (0.84-2.82, p=0.156) | 345 (35.5) | 1.31  (1.11-1.55, p=0.002) | 124 (17.5) | 1.07  (0.83-1.36, p=0.614) | 67 (26.1) | 1.03  (0.68-1.55, p=0.886) |
|  | 3 or more | 50 (9.9) | 1.73  (1.06-2.85, p=0.029) | 346 (15.3) | 1.73  (1.06-2.85, p=0.029) | 635 (35.8) | 1.27  (1.10-1.46, p=0.001) | 284 (17.9) | 1.02  (0.84-1.25, p=0.814) | 153 (21.4) | 0.87  (0.61-1.23, p=0.415) |
| **APACHE II Score** | 0 | 84 (7.6) | Reference | 721 (13.1) | Reference | 1391 (30.9) | Reference | 511 (15.3) | Reference | 263 (23.0) | Reference |
|  | 1-8 | <10 | 0.84  (0.19-2.60, p=0.792) | 18 (17.3) | 0.84  (0.19-2.60, p=0.792) | 27 (23.7) | 0.71  (0.44-1.10, p=0.139) | 21 (23.3) | 1.45  (0.83-2.46, p=0.177) | <10 | 0.55  (0.12-1.77, p=0.363) |
|  | 9+ | 29 (8.8) | 1.38  (0.81-2.30, p=0.228) | 291 (19.2) | 1.38  (0.81-2.30, p=0.228) | 568 (41.6) | 1.05  (0.90-1.23, p=0.504) | 252 (24.8) | 1.56  (1.27-1.92, p<0.001) | 87 (29.2) | 1.44  (1.03-2.02, p=0.034) |
| **Length of hospital Stay** | <7 | 27 (6.8) | Reference | 184 (9.5) | Reference | 319 (20.3) | Reference | 168 (13.6) | Reference | 61 (19.2) | Reference |
|  | 7-13 | 28 (5.5) | 0.85  (0.48-1.52, p=0.578) | 269 (11.1) | 0.85  (0.48-1.52, p=0.578) | 590 (28.9) | 1.50  (1.28-1.77, p<0.001) | 203 (13.3) | 1.12  (0.89-1.42, p=0.338) | 101 (20.2) | 1.24  (0.86-1.81, p=0.258) |
|  | 14+ | 61 (10.7) | 1.94  (1.15-3.32, p=0.014) | 577 (20.9) | 1.94  (1.15-3.32, p=0.014) | 1077 (45.5) | 2.80  (2.39-3.30, p<0.001) | 413 (24.3) | 2.05  (1.65-2.57, p<0.001) | 191 (29.7) | 2.00  (1.40-2.89, p<0.001) |
| **Ventilatory support** | none | 12 (12.2) | Reference | 53 (13.2) | Reference | 101 (30.4) | Reference | 60 (19.7) | Reference | 24 (29.3) | Reference |
|  | non-invasive | 78 (7.8) | 0.46  (0.21-1.03, p=0.050) | 645 (14.4) | 0.46  (0.21-1.03, p=0.050) | 1105 (29.9) | 0.77  (0.58-1.03, p=0.074) | 447 (17.2) | 0.75  (0.53-1.07, p=0.102) | 237 (25.8) | 0.78  (0.45-1.39, p=0.386) |
|  | invasive | 26 (6.8) | 0.41  (0.15-1.18, p=0.095) | 332 (14.8) | 0.41  (0.15-1.18, p=0.095) | 780 (40.0) | 0.64  (0.45-0.90, p=0.010) | 277 (18.0) | 0.67  (0.43-1.05, p=0.081) | 92 (19.9) | 0.52  (0.25-1.08, p=0.076) |
| **Cardiovascular support** | None | 90 (8.2) | Reference | 693 (14.0) | Reference | 1186 (29.3) | Reference | 523 (17.4) | Reference | 253 (26.2) | Reference |
|  | Yes | 26 (6.8) | 0.82  (0.47-1.41, p=0.486) | 337 (15.5) | 0.82  (0.47-1.41, p=0.486) | 800 (41.5) | 1.12  (0.97-1.29, p=0.134) | 261 (18.0) | 0.92  (0.75-1.12, p=0.393) | 100 (20.1) | 0.70  (0.50-0.97, p=0.033) |
| **Renal support** | None | 111 (7.9) | Reference | 914 (13.7) | Reference | 1785 (31.6) | Reference | 712 (17.2) | Reference | 330 (23.8) | Reference |
|  | Yes | <10 | 1.06  (0.34-2.76, p=0.918) | 116 (26.5) | 1.06  (0.34-2.76, p=0.918) | 201 (62.6) | 1.97  (1.51-2.58, p<0.001) | 72 (23.5) | 1.12  (0.81-1.54, p=0.498) | 23 (30.3) | 1.19  (0.64-2.13, p=0.577) |
| **Highest level of support** | none | <10 | Reference | 35 (14.8) | Reference | 41 (21.7) | Reference | 23 (14.7) | Reference | 14 (29.2) | Reference |
|  | level 1 | 20 (8.3) | 1.30  (0.46-4.35, p=0.641) | 131 (13.2) | 1.30  (0.46-4.35, p=0.641) | 239 (27.7) | 1.43  (0.96-2.16, p=0.084) | 119 (19.8) | 1.51  (0.91-2.58, p=0.121) | 64 (27.6) | 0.97  (0.47-2.08, p=0.941) |
|  | level 2 | 67 (8.5) | 1.45  (0.52-4.86, p=0.507) | 479 (13.6) | 1.45  (0.52-4.86, p=0.507) | 825 (28.4) | 1.37  (0.93-2.07, p=0.117) | 321 (16.1) | 1.24  (0.76-2.11, p=0.408) | 163 (23.5) | 0.83  (0.41-1.76, p=0.623) |
|  | level 3 | 24 (6.2) | 0.96  (0.26-3.88, p=0.947) | 385 (16.2) | 0.96  (0.26-3.88, p=0.947) | 881 (43.6) | 2.17  (1.40-3.41, p=0.001) | 321 (18.8) | 1.34  (0.76-2.41, p=0.319) | 112 (23.0) | 1.11  (0.48-2.64, p=0.817) |
| **Surgery** | none | 86 (8.3) | Reference | 739 (14.7) | Reference | 1382 (32.5) | Reference | 588 (18.5) | Reference | 264 (26.9) | Reference |
|  | Elective | 19 (7.2) | 0.88  (0.49-1.51, p=0.650) | 142 (11.9) | 0.88  (0.49-1.51, p=0.650) | 315 (32.0) | 1.18  (1.00-1.39, p=0.043) | 88 (12.0) | 0.75  (0.58-0.96, p=0.025) | 41 (15.3) | 0.57  (0.39-0.83, p=0.004) |
|  | Emergency | 11 (6.4) | 0.75  (0.36-1.43, p=0.404) | 149 (16.3) | 0.75  (0.36-1.43, p=0.404) | 289 (38.9) | 1.22  (1.02-1.45, p=0.030) | 108 (19.7) | 0.94  (0.73-1.19, p=0.593) | 48 (22.9) | 0.72  (0.49-1.05, p=0.090) |

*Note:* Cell values less than 10 are censored. *SIMD:* Scottish Index of Multiple Deprivation; *OR*: odds ratio; *CI*: confidence interval; *APACHE*: Acute Physiology and Chronic Health Evaluation
